# Ruptured Hepatic Artery Aneurysm

**DOI:** 10.7759/cureus.7715

**Published:** 2020-04-17

**Authors:** Amber Mirajkar, Ayanna Walker, Sanjiv Gray, Amanda L Webb, Latha Ganti

**Affiliations:** 1 Emergency Medicine, University of Central Florida College of Medicine/Hospital Corporation of America Graduate Medical Education Consortium of Greater Orlando, Orlando, USA; 2 Emergency Medicine, Osceola Regional Medical Center, Orlando, USA; 3 Surgery, University of Central Florida College of Medicine, Orlando, USA; 4 Emergency Medicine, University of Central Florida College of Medicine, Orlando, USA; 5 Emergency Medicine, Envision Physician Services, Nashville, USA; 6 Emergency Medical Services, Polk County Fire Rescue, Bartow, USA

**Keywords:** hepatic artery aneurysm

## Abstract

Aneurysmal ruptures are a life-threatening pathology, and while the aorta is the principal location, any aneurysmal rupture can be fatal. Most result from chronic diseases, such as hypertension, diabetes, and vasculitis. Nevertheless, a rupture can result in acute decompensation and must be recognized and addressed quickly to limit morbidity and mortality. The authors describe a case of a 66-year-old female who presented to the emergency department (ED) for abdominal pain and syncope. Even though imaging did not explicitly show the specific site of rupture of the hepatic artery, the positive Rapid Ultrasound for Shock and Hypotension (RUSH) exam and aortic dissection on computed tomography angiography along with her clinical picture (hypotension, abdominal pain, decreased capillary refill, grey skin) raised our suspicions for critical pathology. Exploratory laparotomy revealed a ruptured hepatic artery aneurysm. Her hospital course was complicated by ischemic necrosis of the gallbladder, spleen, and liver, requiring cholecystectomy, splenectomy, and partial hepatectomy, but she was discharged to rehabilitation and expected to make a recovery. This case displays the importance of using ultrasonography early to aid in expedited diagnosis and treatment as well as maintaining a high suspicion for vascular pathology in the setting of hemorrhagic shock.

## Introduction

With the population getting sicker and the baby boomers aging, the number of people with aneurysms and aortic dissections is increasing. Although a rare type of aneurysm with an overall prevalence of 0.002-0.4%, hepatic aneurysms make up about 20% of visceral aneurysms and have a 44% rate of rupture [1-2]. These aneurysms usually have accompanying co-morbidities. Just like with aortic aneurysms, risk factors for visceral aneurysms (such as a hepatic artery aneurysm) include atherosclerosis, medial degeneration infection, trauma, and vasculitides [3-5]. As those with complex and concurrent medical problems age, so do their chances of complications like these increase. 

Although abdominal aortic aneurysm (AAA) rupture is commonly on the differential for abdominal pain in the older population, there can be other aneurysmal ruptures, especially in those with vascular disease. Even if computed tomography angiography (CTA) does not explicitly show the source of clinical hemorrhagic shock, one’s suspicion for vascular compromise must be maintained.

## Case presentation

A 66-year-old female presented to the emergency department (ED) via emergency medical services (EMS) with a chief complaint of abdominal pain and syncope. Per EMS, the patient syncopized upon their arrival but regained consciousness shortly thereafter. Pre-hospital management included 250 cc of normal saline (NS) and 2 L of oxygen via nasal cannula. For EMS, glucose was 108 mg/dL, respiratory rate 22 breaths/minute, oxygen saturation 94% on room air, and blood pressure 127/72 mmHg. The patient was not able to communicate her full history at the time as her mental status waxed and waned. Per the daughter, the patient started complaining of abdominal pain 12 hours prior to arrival. The pain became progressively worse and was described as sharp, constant, diffuse, and rated 10/10. The daughter called 911 upon arriving home from work and finding her mother weak and pale. Medical history per daughter included repair of a dissecting Type A thoracic aneurysm (TEVAR with Gore thoracic endograft 40 x 15) 4 months prior, descending aortic dissection (from graft to celiac artery), infrarenal aortic aneursym, L internal iliac artery aneursym, congestive heart failure, chronic obstructive pulmonary disease, hypertension, diabetes mellitus type II, and atrial fibrillation on warfarin. Other medications included lisinopril, metoprolol, amiodarone, furosemide, metformin, potassium, atorvastatin, and flucticasone/salmeterol.

Upon arrival to the ED, the patient was cool, pale, diaphoretic, and soporific, however, oriented x3 when aroused. The Glasgow coma score (GCS) was 14 (E3V5M6). She was mildly hypotensive with a blood pressure of 101/59 mmHg, afebrile, tachypneic, and with a normal pulse rate of 80 beats per minute. The patient's physical exam findings were remarkable for an abdomen that was soft, diffusely tender, without rebound, guarding or palpable mass, and decreased bowel sounds. 

After establishing intravenous (IV) access and initiating fluid resuscitation with NS, the rapid ultrasound for shock and hypotension (RUSH) exam was performed within minutes of the patient's arrival [6-9]. A large hypoechoic infra-umbilical fluid collection was revealed upon visualization of the suprapubic window. The aorta views were inadequate secondary to a technically difficult examination, but the patient's splenorenal and Morrison's pouch views were negative for acute findings.

Given the initial presentation of syncope, worsening hypotension (90/61 on repeat), diaphoresis, conjunctival pallor, and delayed capillary refill, our biggest concern was for hemorrhagic shock. Although unsure of the source, the differential included but was not limited to AAA rupture, aortic dissection, aortoenteric fistula, compromise of the ascending aorta elephant trunk graft, diverticulitis, perforated ulcer, perforated diverticulitis, mesenteric ischemia, perforated gallbladder, sepsis, volvulus, and any other state which causes vascular compromise. We could not rule out sepsis, and so the patient was concurrently started on 2L normal saline, vancomycin, and cefepime.

**Figure 1:**
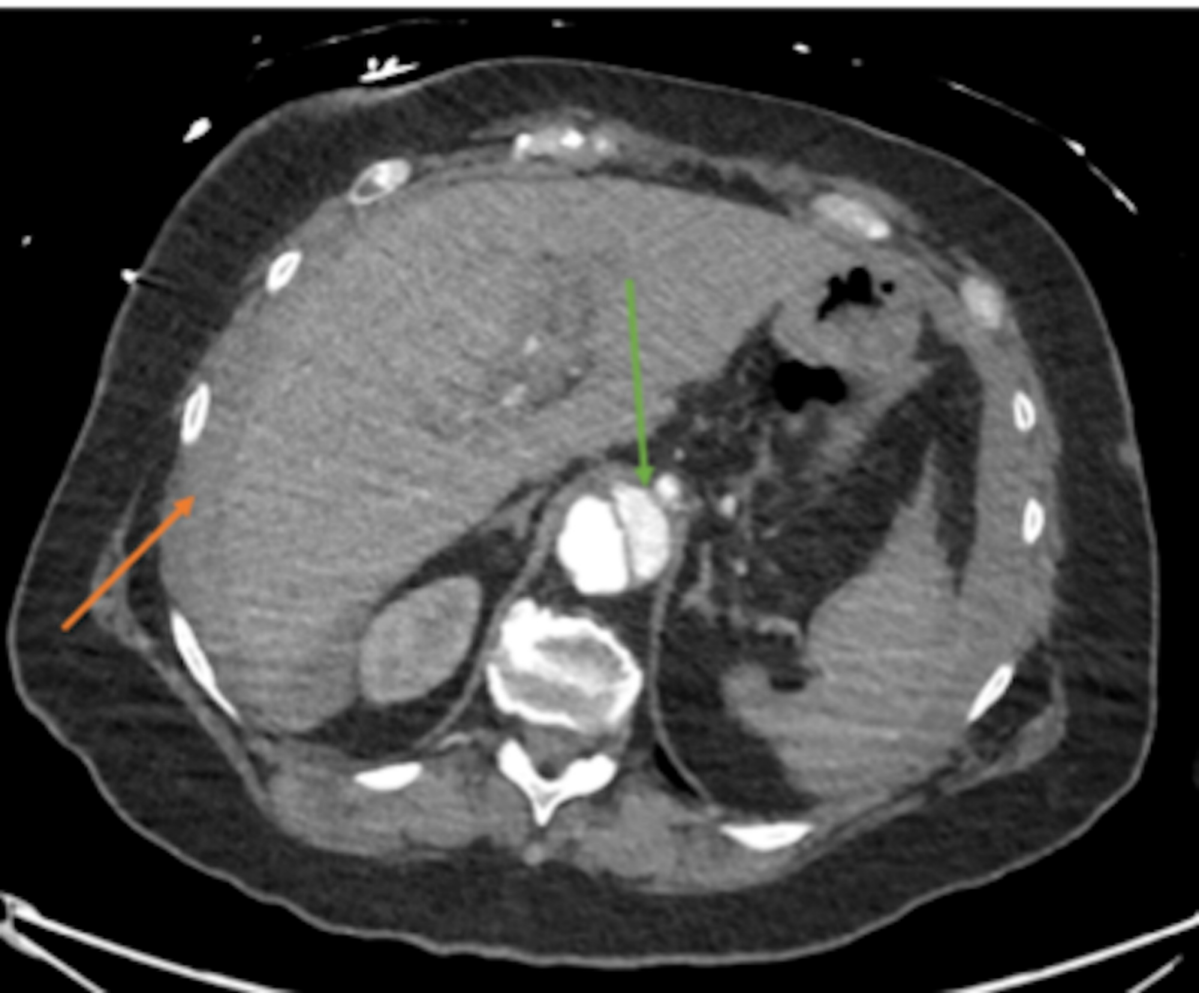
Existing aortic dissection feeding the celiac trunk (green arrow); hypoechoic capsule seen around liver, thought to be extravasated blood (orange arrow)

**Figure 2:**
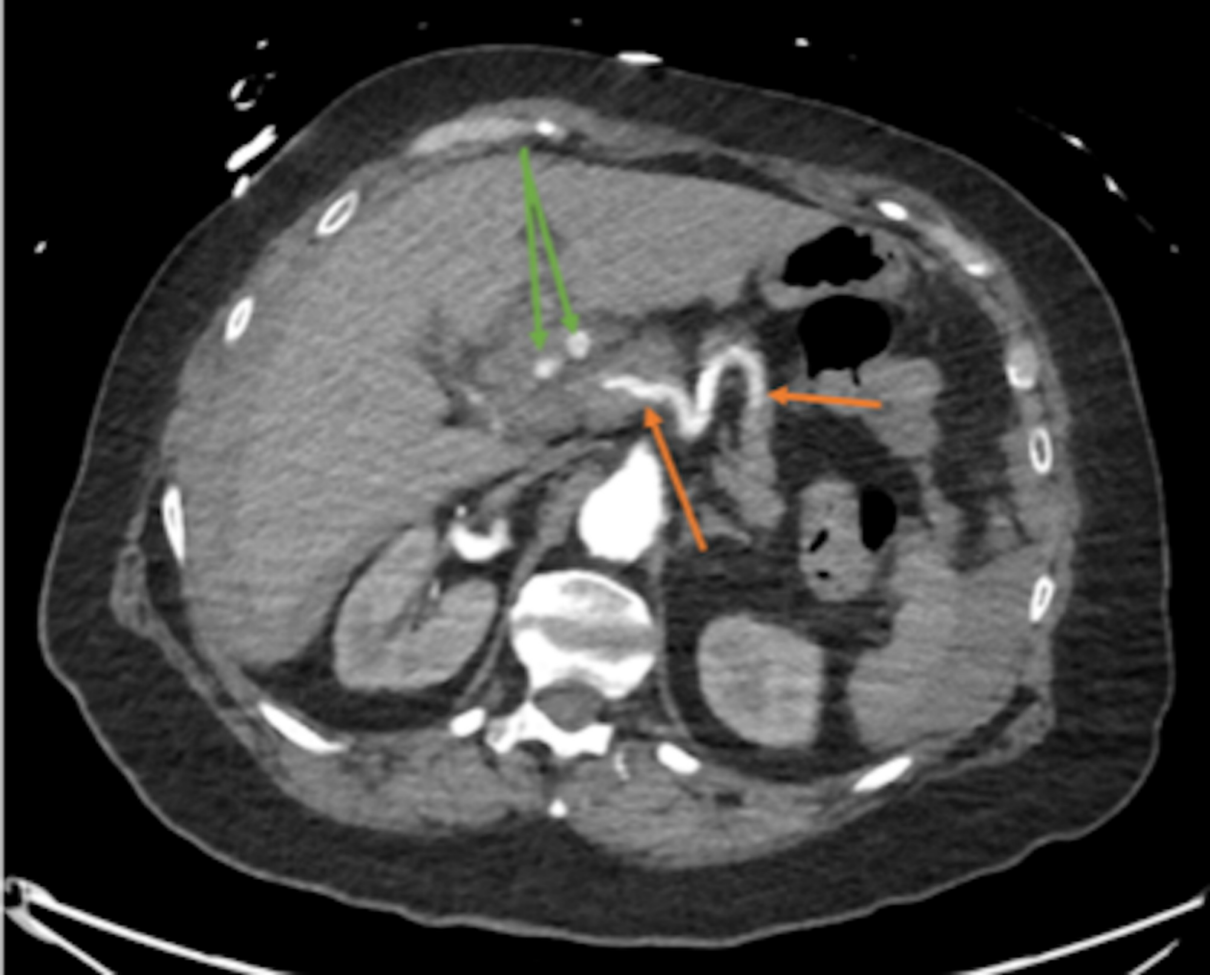
Filling of left gastric and common hepatic arteries (orange arrows); downstream filling of 2 out of the 3 branches of the common hepatic arteries (green arrows); aneurysmal disease in the right renal artery

**Figure 3:**
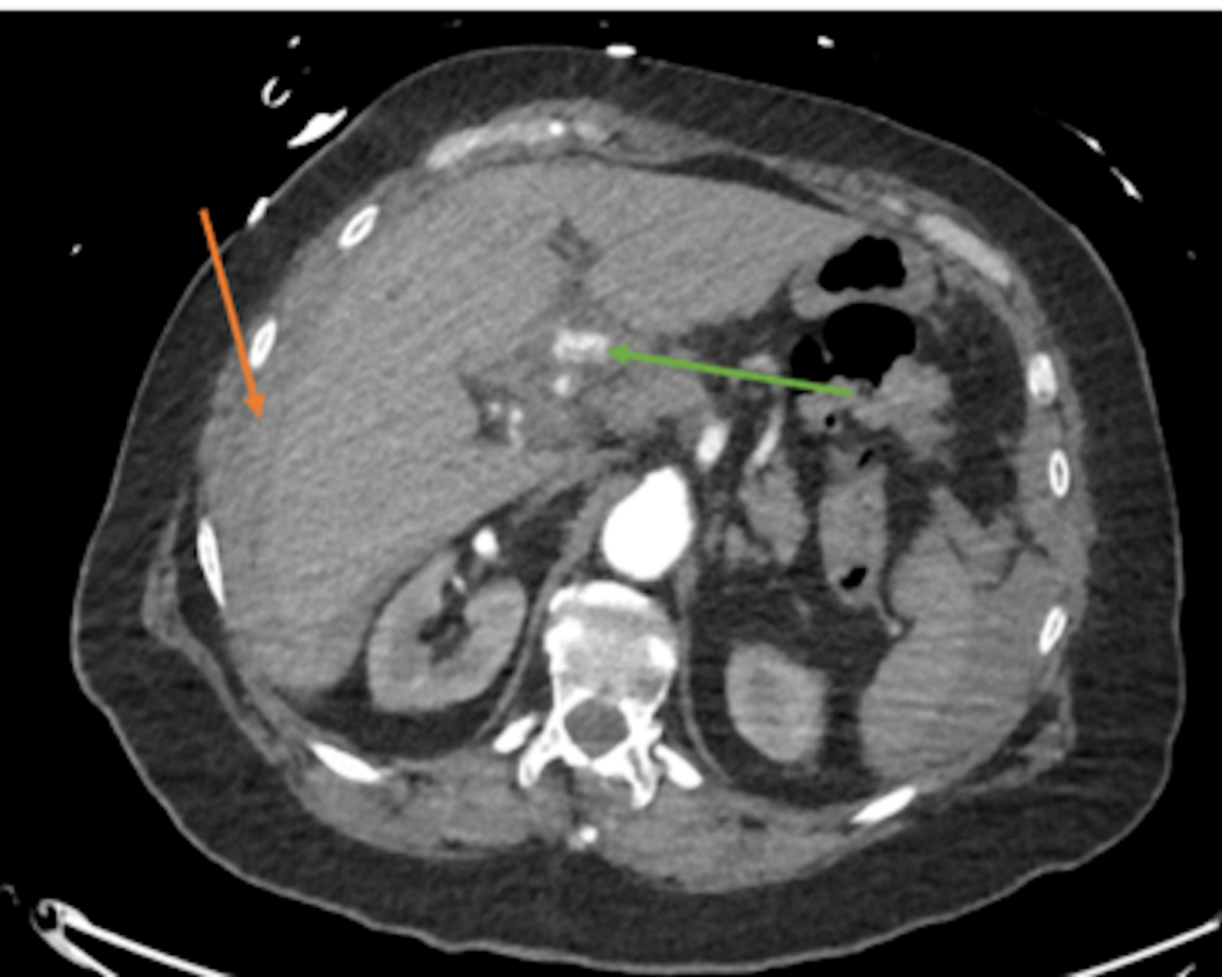
Green arrow demonstrates possible proper hepatic artery aneurysm measuring 9.78 mm (normal: 4-6 mm), but without obvious extravasation Presence of hypoechoic capsule around the liver, possibly indicating older blood (orange arrow). This finding in the light of a +FAST and clinical picture is what prompted general surgery to take the patient for exploratory laparotomy.

**Figure 4:**
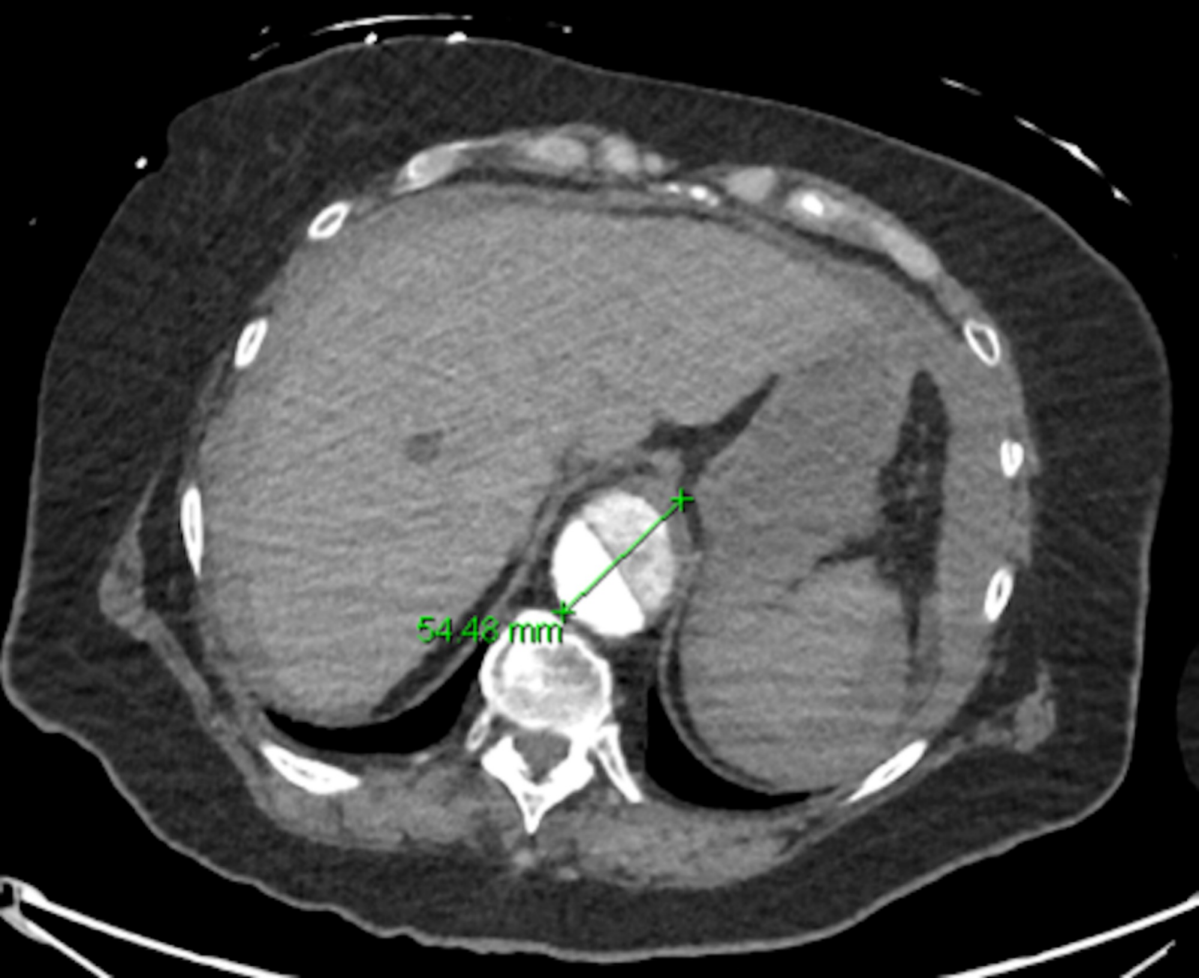
Suprarenal aortic aneurysm with concurrent dissection

**Figure 5:**
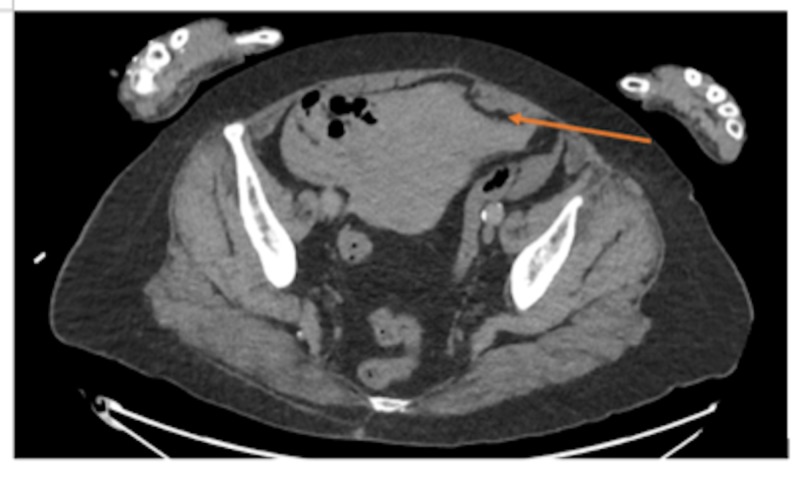
Free fluid in the pelvis (arrow)

As the patient became progressively more hypotensive (80s/60s) and multiple attempts at second peripheral IV access failed, a central line was placed for emergency release blood. A chest radiograph and CTA of the chest, abdomen, and pelvis were obtained. The patient was found to have supra- and infrarenal abdominal aortic aneurysms, the infrarenal aneurysm measuring 3.5 cm x 3.4 cm with intimal dissection and extension into the common iliac arteries. There was also a dissection of the abdominal aorta extending from the aortic graft into the celiac, superior mesenteric, and renal arteries, with the false lumen feeding the celiac artery. Finally, CTA showed a complex infraumbilical ascites or blood collection, measuring 8 cm x 10 cm x 7 cm. No artery aneurysm rupture or active extravasation was seen (Figures 1-4). The suprarenal aneursym, measuring 5.4 cm x 5.1 cm, was new compared to her imaging 4 months prior, as was the fluid collection (Figure 5).

Abnormal labs were as follows: international normalized ratio (INR) 2, point of care (POC) lactic acid 7.9 mmol/L, hemoglobin 9.9 gm/dL, hematocrit 30%, calcium 7.9 mg/dL, potassium 2.9 mmol/L, and CO_2_ 20 mmol/L. 

Surgical consultation was obtained. Although the CTA abdomen/pelvis could not clearly show the HAA, our general surgeon noted blood in the hepatic capsule, and in the pelvis (Figure 5), which was concerning to him. Given the extensive aneurysms and dissections of the thoracic, abdominal, and pelvic arterial systems, the suspicion was still very high for a vascular rupture. In the ED, the patient received 4 units of packed red blood cells, 2 units of fresh frozen plasma, 1 dose of anti-inhibitor coagulant complex (FEIBA), 10 mg of vitamin K (phytonadione), and 10 mg of metoprolol IV. After examining the patient, reviewing labs and imaging, and seeing the trended vitals, general surgery agreed to take the patient to the OR, with vascular surgery on stand-by.

In the operating room, general surgery and vascular surgery found a ruptured hepatic artery aneursym with approximately 2 L of hemoperitoneum. The proper hepatic artery was ligated proximally, excised, and abdomen packed. The patient received an additional unit packed red blood cells and unit of platelets, for a total of 5 units packed RBCs, 2 units of FFP, and 1 unit of platelets. The abdomen was temporarily closed with a wound vac for further resuscitation and planned second surgery for celiac artery bypass. The following day, she was taken back to the OR for the vascular procedure; however, marked ischemia was noted to the gallbladder and hepatic segments 2, 3, 4a, and 4b. The patient underwent emergent cholecystectomy but was not able to undergo a celiac artery bypass. The abdomen was temporarily closed with a wound vac for further resuscitation and planned third look surgery. On day 3, the patient was again taken back to the operating room but hepatic segments 2 and 3 had infarcted; non-anatomical left partial hepatectomy including segments 2 and 3 was performed. The abdomen was temporarily closed with a wound vac for further resuscitation and planned fourth look surgery. On day 4, the patient returned to the operating room and underwent splenectomy secondary to ruptured splenic hematoma and splenic necrosis along with fascial closure and incisional wound vacuum. The patient was extubated on hospital day 7. On day 19, the patient was ambulatory and discharged to rehab. Complications in the hospital included hospital-acquired pneumonia, acute kidney injury, deep vein thromboses in the right upper extremity, and a poorly-healing surgical wound. She was sent home with a wound vacuum, IV antibiotics. and oxygen (a new requirement). The patient was started on Eliquis at discharge, replacing her warfarin. No blood pressure targets were listed in the discharge summary; however, the patient was to follow up with cardiology, vascular surgery, and general surgery outpatient. She was able to see general surgery 2 weeks after discharge but was in guarded condition. The aneursyms and dissections were being managed medically. The patient was seen in the clinic after 3 months with healed wounds and is back to her usual activities.

## Discussion

Hepatic artery aneurysms (HAA) and other visceral aneurysms are a rare, but serious, condition. Renal artery aneurysms are the most common visceral aneurysms, followed by splenic, then hepatic in descending prevalence. In one meta-analysis evaluating 80 cases, 359 out of 2845 visceral aneurysms were hepatic (12%), with an incidence of 0.01 to 0.2% [3,6]. HAAs are approximately 20% of splanchnic aneurysms and are at risk of rupturing when diameter exceeds 2 cm [10-11] There have not been enough cases to determine the best way to repair them - i.e. open vs. endovascularly. The vast majority of publications are case reports and have similar outcomes regardless of approach. Given the high rate of rupture, 44%, repair is recommended [4]. Endovascular techniques offer a less invasive approach and faster recovery. The utilization of endovascular management is increasing [12-13]. However, these patients are usually stable and fewer co-morbidities than our patients. Unfortunately, given our patient's hemorrhagic shock seen on physical examination and labs, her extensive co-morbidities, and that the aneurysm had already ruptured, she was not a good candidate for endovascular repair. Additionally, the ruptured hepatic artery aneurysm was only diagnosed on exploratory laparotomy. Interestingly, pseudoaneurysms have an even higher risk of rupture [1,11].

HAA can be further classified into HAA and giant HAA, with giant HAA (>5 cm) being very rare [14]. Given that the HAA was not discovered until ruptured in our patient, it is unclear if ours was giant or not. Most HAAs are discovered when they rupture, and subsequently, patients become symptomatic or are found incidentally when imaged for other purposes [15]. Unfortunately, once they rupture, maintaining perfusion to the hepatobiliary organs can be difficult [16]. Furthermore, depending on where the rupture is and subsequent ligation (i.e. proximal or distal to the gastroduodenal artery), necrosis can occur quickly [17]. As in our patient who was not able to have a celiac artery bypass after ligation of her proximal proper hepatic artery, this often requires resection of the gallbladder, liver, and/or spleen. The patient is at risk for biliary strictures and hepatitis and should she be seen again in the ED.

Definitive measures for intact or ruptured HAA, given the rarity of the disease, are not standardized [1,6,10]. However, it is reasonable to treat this pathology similar to an intact or ruptured aortic aneursym - decrease the shearing forces with beta-blockers. The patient was on metoprolol outpatient, and we continued to this therapy to lower to HR in the ED. However, given her hypotension and shock state, there was a concern for hypoperfusion if we decreased the cardiac output too much. Nevertheless, this patient and others like her will need strict outpatient follow-up for blood pressure and heart rate control, lest this happens again to her other aneurysms.

Similar to a ruptured AAA, a ruptured visceral artery is a surgical emergency but often presents with nausea, vomiting, and abdominal pain - the vague constellation of symptoms seen all too frequently in a busy ED. Regrettably, many visceral artery aneurysm cases have resulted in high morbidity and mortality because they are often caught after rupture, similar to our patient. Unfortunately, there is no screening for them the way the US Preventive Services Task Force screens for abdominal aortic aneurysms in men 65 to 75 who have ever smoked with a one-time abdominal aortic ultrasound [18]. What is key for ED providers is to recognize clinical hemorrhagic shock. Once there is a concern for hemorrhagic shock in a patient with a history of arterial aneurysms and dissections, the suspicion for a rupture of one of these structures, seen or unseen, must remain high to prevent morbidity and mortality.

## Conclusions

In our case, the patient had a history of aortic dissections and aneurysms, including one previous graft surgery in the ascending thoracic aorta and aortic arch. So, it is not surprising these pathologies extended into the medium-sized vessels. This important history coupled with the clinical picture of hemorrhagic shock is largely why our suspicion for an aneurysm rupture was so high, even if we could not find the location easily on CTA. The key point for ED providers is to continue to have a high suspicion for aneurysmal or dissection rupture in patients like ours, even with inconclusive imaging, if the clinical picture is one of hemorrhagic shock.
